# Quercetin with the potential effect on allergic diseases

**DOI:** 10.1186/s13223-020-00434-0

**Published:** 2020-05-14

**Authors:** Morteza Jafarinia, Mahnaz Sadat Hosseini, Neda kasiri, Niloofar Fazel, Farshid Fathi, Mazdak Ganjalikhani Hakemi, Nahid Eskandari

**Affiliations:** 1 Department of Immunology, Faculty of Medicine, Isfahan University of Medical Sciences, Box 8174673461, Isfahan, Iran; 2 Applied Physiology Research Center, Isfahan Cardiovascular Research Institute, Department of Physiology, School of Medicine, Isfahan University of Medical Sciences, Isfahan, Iran

**Keywords:** Quercetin, Allergy, Asthma

## Abstract

Quercetin is a naturally occurring polyphenol flavonoid which is rich in antioxidants. It has anti-allergic functions that are known for inhibiting histamine production and pro-inflammatory mediators. Quercetin can regulate the Th1/Th2 stability, and decrease the antigen-specific IgE antibody releasing by B cells. Quercetin has a main role in anti-inflammatory and immunomodulatory function which makes it proper for the management of different diseases. Allergic diseases are a big concern and have high health care costs. In addition, the use of current therapies such as ß2-agonists and corticosteroids has been limited for long term use due to their numerous side effects. Since the effect of quercetin on allergic diseases has been widely studied, in the current article, we review the effect of quercetin on allergic diseases, such as allergic asthma, allergic rhinitis (AR), and atopic dermatitis (AD).

## Background

Medicinal plants are become more popular in the last decades regard to their low price, natural origin, and fewer side effects [1]. Studies indicated several plant-derived secondary metabolites that can down-regulate the expression and production of inflammatory mediators and their receptors and inhibit the expression of transcription factors which are promoting the secretion of mediators [2]. Researchers are attributing quercetin as one of the well-known types of plant’s metabolites [2]. Quercetin (3,31,41,5,7-pentahydroxyflavone), a naturally occurring polyphenol flavonoid, found in some fruits and vegetables, including onions, capers, apples, berries, tea, tomatoes, grapes, Brassica vegetables, and shallots, as well as many nuts, seeds, barks, flowers, and leaves [3, 4]. The highest concentration of quercetin is 234 mg/100 g of edible portion in capers (raw) and the lowest concentration is 2 mg/100 g of edible portion in black or green tea (*Camellia sinensis*) [5].

**Fig. 1:**
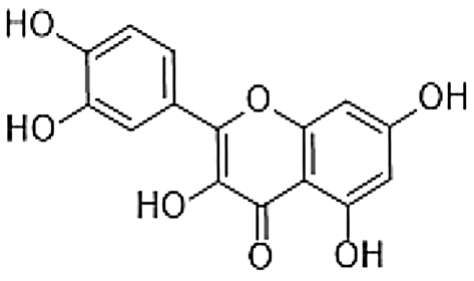
Structure of quercetin

Therefore, quercetin is one of the main flavonoids in our diet and our body requires between 5 and 40 mg daily uptakes of it. Quercetin is largely metabolized in the intestine and liver. The plasma level of quercetin is normally in low ranges, but after consuming foods that are highly rich in it, the plasma’s level of it increase to different ranges. Quercetin contains 3 rings and 5-hydroxyl group (Fig. 1) and it is naturally found in plants as a glycone or carbohydrate conjugates [6–8].

There are several potential benefits of total health and disease resistance such as anti inflammatory, antioxidant and also the ability to inhibit lipid peroxidation, platelet aggregation and capillary permeability [9]. Anti-inflammatory effect of quercetin has been shown in several studies. Quercetin inhibits lipopolysaccharide (LPS)-induced tumor necrosis factor alpha (TNF-α) production in macrophages [10], LPS-induced interleukin (IL)-8 production in lung A54 cells [11], LPS-induced mRNA levels of TNF-α and IL-1α in glial cells [12], production of inflammation-producing enzymes (cyclooxygenase (COX) and lipoxygenase (LOX) [13], and FcεRI-mediated release of proinflammatory cytokines, tryptase, and histamine from human umbilical cord blood-derived cultured mast cells [14]. Based on different effects of quercetin, we review the effect of quercetin on allergic asthma, allergic rhinitis (AR), and atopic dermatitis (AD) in this article.

## Allergic asthma

Asthma is a chronic inflammatory lung disease. The typical signs of asthma include airway obstruction, wheezing, and airway hyperresponsiveness. It characterized by airway hyperresponsiveness to allergens, airway edema, increased mucus secretion by the recruitment of eosinophils and other leukocytes, airway smooth muscle hypertrophy/hyperplasia, systemic immunoglobulin E (IgE) production and mucus hypersecretion [15–18]. The etiology of asthma is multifactorial and it seems that combinations of genetic and environmental factors are involved. Allergic asthma is mediated by CD4^+^ T cell immune reactions. T helper (Th) 1 and Th17 cells increase neutrophil recruitment and Th9 cells effectively change the mucus production, mast cell recruitment, and IgE production. CD8^+^ T cells, NKT cells, and ɣδ T cells are also able to modulate asthma associated inflammation and/or airway hyperresponsiveness. T regulatory (Treg) cells are known as innate suppressors and adaptive immune responses and also the factors for reducing inflammation. Th2 immune response is one of the most essential factors that are linked with the pathology of asthma. Cytokines produced by the Th2 cells in asthma (IL-4, IL-5, IL-13) have a critical role in the inflammatory response. Cytokines, including IL-4, stimulate B cells to synthesize IgE, IL-13, and IL-5 which are required for eosinophilic access to the lung tissue in order to increase vascular permeability and chemotaxis, that can improve the inflammatory response. Besides, activated mast cells are able to release inflammatory and bronchoconstrictor mediators during an allergen challenge [19–21]. Most allergens or helminth-antigen-specific human T CD4^+^ cell clones exhibit a Th2 phenotype. In allergic diseases, Th1/Th2 balance shifts to Th2 phenotype [22]. History of atopic diseases is the strongest risk factor for developing asthma. People suffering from hay fever or eczema are more probable to allergic asthma. Environmental triggers for asthma include exercise, pollutants, hyperventilation, and hormonal change. Both indoor and outdoor allergens and pollutants including biological allergens (dust mites, cockroaches, mold, and animal dander), irritant chemicals, fumes-traffic pollution, high ozone level, and product by combustion device are important [23].

Asthma treatment is based on inhaled corticosteroids, β2-agonists, anti-cholinergic, and methylxanthines. While using mentioned drugs alone or as a combination has a great effect, some side effects limit their usage. Thus, it is essential to develop new compounds having similar therapeutic potential and less adverse effects on the constant treatment of respiratory diseases [19]. In the following, the in vitro and in vivo effects of quercetin on allergic asthma are reviewed.

### Effect of quercetin on allergic asthma

#### In vitro studies

Most studies have investigated the impact of quercetin on tracheal tissue contractility and mucin 5 AC (MUC5AC). In 2009, the effect of quercetin on isolated tracheal tissue from male Wistar rats has been evaluated by Capasso et al. They showed quercetin (in concentrations of 10^−6^ – 3 × 10^−4^ M) inhibits rat tracheal contractility through a presynaptic and a postsynaptic site of action. They concluded that quercetin could be considered as a possible application in airways diseases such as asthma [24]. Two studies have been investigated the effect of quercetin on MUC5AC in allergic asthma. In 2010, Chang et al. indicated the dietary polyphenols ([6]-gingerol, epigallocatechin gallate (EGCG), curcumin, and quercetin) inhibit MUC5AC gene expression in NCI-H292 cells, and also their effect on ciliary beat frequency (CBF) of the human nasal mucosa. The minimum inhibitory concentration of MUC5AC in quercetin was 40 µM. They concluded that Gingerol, quercetin, and EGCG may be considered as anti-hypersecretory agents because they effectively inhibit mucus secretion of respiratory epithelial cells while maintaining normal nasal ciliary movement [25]. In the same year, the effect of quercetin on MUC5AC expression induced by human neutrophil elastase (HNE) in human airway epithelial (HBE16) cells and its molecular mechanisms has been investigated by Li et al. They pretreated HBE16 cells with quercetin and also treated with HNE. Their results suggest that quercetin can inhibit HNE-induced MUC5AC expression in human airway epithelial cells through protein kinase C (PKC)/epidermal growth factor receptor (EGFR)/extracellular-regulated kinase (ERK) signal transduction pathway. The significant inhibitory dose for quercetin was 40 µM [26] (Table 1). In vitro studies of quercetin suggest that quercetin in the concentration of 40 µM is useful for mucus hypersecretion, a common pathological change in chronic inflammatory diseases of the airway. In the future, quercetin might be a valuable treatment for mucin hypersecretion in chronic inflammatory airway diseases in the clinic.

**Table 1:** Summary of main effects of quercetin on allergic diseases (in vitro studies)

Diseases	Dosage	Cell/cell line	Animal	Quercetin’s effect	References
Allergic asthma	10^−6^–3 × 10^−4^ M	Tracheal tissue	Male Wistar rat	Inhibits rat tracheal contractility	[24]
	40 µM	NCI-H292 cells	n/a	Inhibit MUC5AC gene expression	[25]
	40 µM	HBE16	n/a	Inhibit HNE-induced MUC5AC expression in human airway epithelial cells through PKC/EGFR/ERK signal transduction pathway	[26]
Allergic rhinitis	4.0 μM	HNEpC	n/a	Suppressed the ability of cells to produce RANTES and eotaxin	[38]
	1.0 nM	HNEpC		Modified the clinical condition of AR through the suppression of NO production from nasal epithelial cells after IL-4 stimulation	[41]
Atopic dermatitis	30 μM	RBL-2H3	Wistar rat and BALB/c	Upregulation of HO after short exposure Induction of HO-1 expression after long exposure	[51]
	5 mg/ml	Keratinocyte	n/a	Strong ability to scavenge free radicals and protect human keratinocytes against hydrogen peroxide damage	[52]

*MUC5AC* mucin 5 AC, *HNE* human neutrophil elastase, *PKC* protein kinase C, *EGFR* epidermal growth factor receptor, *ERK* extracellular-regulated kinase, *RANTES* regulated on activation, normal T cell expressed and secreted, *NO* nitric oxide, *RA* allergic rhinitis, *HO* heme oxygenase, *HNEpC* human nasal epithelial cells

#### In vivo studies

Most of the in vivo studies have focused on the effects of quercetin on immunological aspects of asthma, such as cytokine levels, recruitment of leukocytes, and regulation of Th1/Th2 balance.

Two studies have been investigated the effect of quercetin in the immediate phase response (IAR) and late-phase response (LAR) of allergic reactions. In 2007, the effects of quercetin and rutin on asthmatic responses which were studied in ovalbumin (OVA)-sensitized conscious guinea-pigs challenged with aerosolized-OVA (aOVA) have been considered by Jung et al. Quercetin and rutin inhibited the specific airway resistance (sRaw) in LAR and IAR dose-dependently, as well as the recruitment of leukocytes, particularly eosinophils and neutrophils on LAR. Also, quercetin and rutin (7.5 mg/kg) inhibited sRaw and leukocyte recruitment at a similar level as dexamethasone in LAR and salbutamol in IAR. Thus, they indicated quercetin and rutin may be useful in the treatment of IAR and LAR in asthma [21]. The effects of quercetin inhalation on IAR, LAR and late LAR (LLAR) asthmatic responses with exposure to aOVA which were studied in conscious guinea-pigs sensitized with aOVA have been investigated by Moon et al. Quercetin (10 mg/ml) significantly decreased histamine and protein contents, phospholipase (PL) A2 activity, and recruitments of leukocytes in bronchoalveolar lavage fluid (BALF) and also slightly increased infiltration of eosinophils and neutrophils in the histopathological survey. Quercetin’s anti-asthmatic activity was similar to cromolyn sodium and dexamethasone [27].

**Fig. 2:**
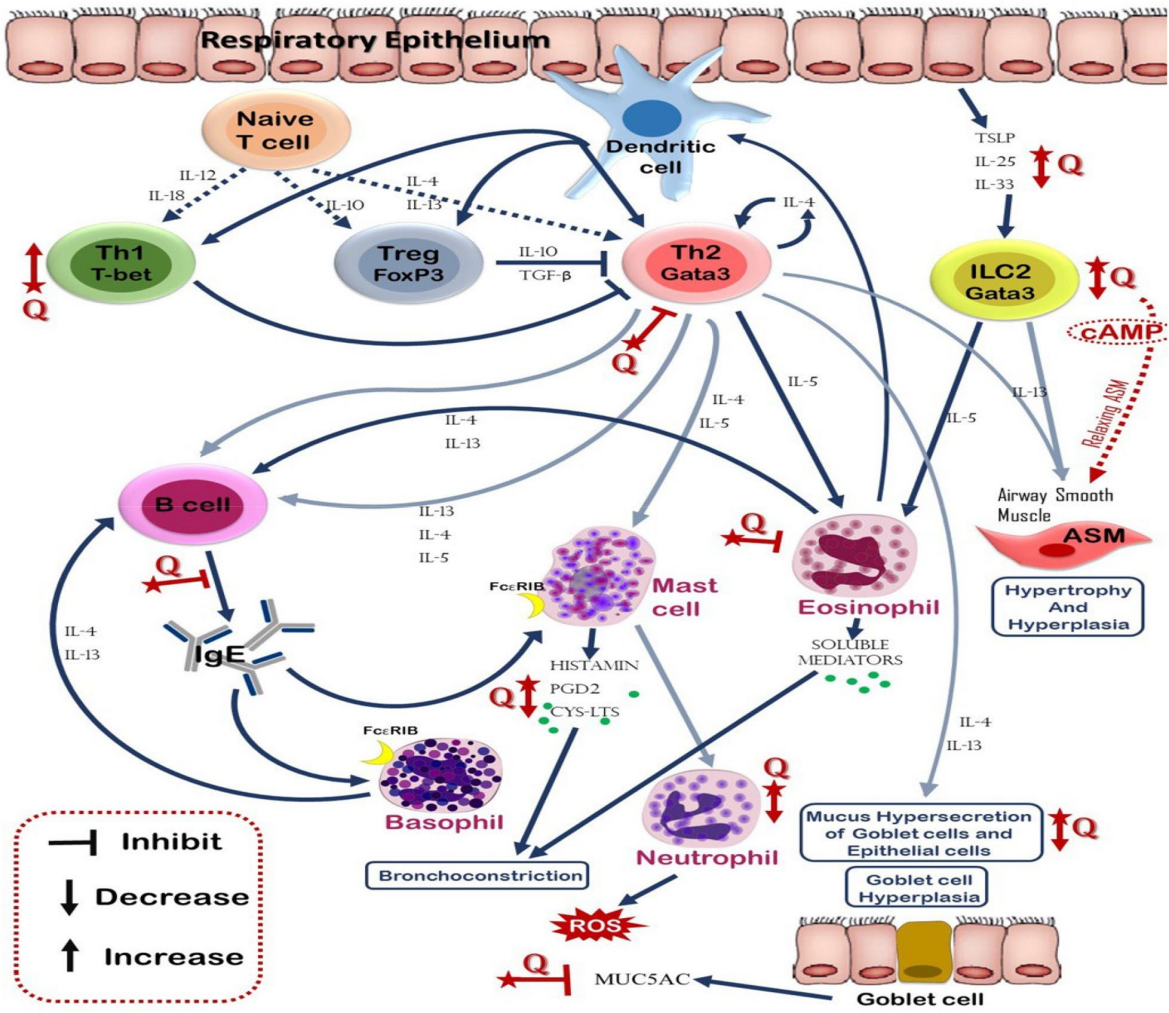
Different effects of quercetin on immune cells involved in asthma

In allergic diseases, Th1/Th2 balance shifts to Th2 phenotype, so investigation on the effect of quercetin on Th1/Th2 balance seems to be essential. In 2009, quercetin’s role in regulation of Th1/Th2 balance and cytokine production, T-box protein expressed in T cells (T-bet) and GATA-3 gene expression in OVA-induced asthma model mice (BALB/c mice) has been studied by Park et al. Mice were injected intraperitoneal with 8 or 16 mg/kg/day in 200 µl of quercetin each day. Results strongly indicated that quercetin decreased allergic airway inflammation and hyperresponsiveness due to the alteration of Th1/Th2 differentiation via the suppression of GATA-3 and the increase of T-bet expression. They also showed that quercetin reduced the increased levels of IL-4, increased interferon (IFN)-ɣ, and significantly inhibit all asthmatic reactions. They suggested that quercetin might be a new therapeutic line to the allergic airway diseases (Fig. 2) [20]. In 2008, Zhu et al. studied *Elaeagnus pungens* leaf that confirming to be very operative for the treatment of asthma and chronic bronchitis as traditional Chinese medicine. Quercetin seems to be the major effective components of *Elaeagnus pungens* leaf. They suggested that quercetin and kaempferol are effective in treating asthma and chronic bronchitis [28]. There were also some other studies that investigated on the leukocyte counts and cytokine levels in BALF. The study of Rogerio et al. in 2007 have shown that in BALB/c receiving quercetin (10 mg/kg), eosinophil counts were lower in BALF, blood and lung parenchyma [29]. The same group in 2010 compared the anti-inflammatory effects of quercetin-loaded microemulsion (QU-ME) and quercetin suspension (QU-SP) in an experimental model of airways allergic inflammation. Mice received a daily oral dose of QU-ME (3 or 10 mg/kg) or QU-SP (10 mg/kg). Their results showed QU-ME reduced the eosinophil recruitment, IL-4 and IL-5 levels in the BALF, as well as, inhibited the nuclear transcription factor-kappa B (NF-κB) activation, P-selectin expression and the mucus production in the lung. As the plant-derived flavonoid quercetin is part of many foods and seems to be safe despite long-term use in animals and humans, therefore, its microemulsion would form an interesting and practical formulation to increase its oral bioavailability and, in turn, to evaluate its potential clinical advantage for treating certain inflammatory and allergic diseases [17]. In 2016, the influence of quercetin (16 mg/kg/day) on histopathological aspects and also airway epithelium in allergic airway inflammation on BALB/c mice has been evaluated by Sozmen et al. Quercetin treatment cause lower epithelial thickness, subepithelial smooth muscle thickness, goblet, and mast cell numbers compared to untreated mice with allergic airway inflammation. However, quercetin treatment was not effective in improving basal membrane thickness. Immunohistochemical scores of IL-25, IL-33, Thymic stromal lymphopoietin (TSLP), cysteine-dependent aspartate-specific proteases (caspase)-3 and terminal deoxynucleotidyl transferase-mediated dUTP nick end labeling (TUNEL) were lower in quercetin-treated mice in comparison with untreated mice with allergic airway inflammation. IL-4, IL-25, IL-33, TSLP levels in BALF and OVA-specific IgE in serum were lower in quercetin treated mice compared with untreated mice. These results suggest that quercetin improves chronic histopathological changes except for basal membrane thickness in lung tissue and its beneficial effects on inflammation might be related to epithelium-derived cytokines modulators and epithelial apoptosis [31] (Table 2). Similar to in vitro studies, in vivo studies suggest that quercetin plays a critical role in asthmatic reactions. Anti-inflammatory effects of quercetin such as reduction of IL-4 and IgE in serum could be useful on allergic asthma.

**Table 2:** Summary of main effects of quercetin on allergic diseases (in vivo studies)

Diseases	Dosage	Animal	Quercetin’s effect	References
Allergic asthma	7.5 mg/kg	Male Dunkin-Hartley guinea pigs	Inhibited the sRaw in LAR and IAR dose-dependently, as well as the recruitment of leukocytes, particularly eosinophils and neutrophils on LAR	[21]
	10 mg/ml	Male Dunkin-Hartley guinea pigs	Decreased histamine and protein contents, PLA2 activity, and recruitments of leukocytes in BAL fluid and also slightly increased infiltration of eosinophils and neutrophils in histopathological survey	[27]
	8 or 16 mg/kg	BALB/c	Decreased allergic airway inflammation and hyperresponsiveness due to the alteration of Th1/Th2 differentiation via the suppression of GATA-3 and increase of T-bet expression Reduced the increased levels of IL-4, increased IFN-ɣ, and significantly inhibit all asthmatic reactions	[20]
	10 mg/kg	BALB/c	Reduced eosinophil counts in BALF, blood and lung parenchyma	[29]
	10 mg/kg	BALB/c	Reduced the eosinophil recruitment, IL-4 and IL-5 levels in the BALF, as well as, inhibited the NF-κB activation, P-selectin expression and the mucus production in the lung	[17]
	5 mg/kg	Sprague–Dawley rats	Diminished the increase of total cell numbers and macrophage numbers in BALF Decreased the neutrophil and lymphocyte counts, levels of TNF-α, IL-1β, and IL-6	[30]
	16 mg/kg	BALB/c	Lower epithelial thickness, subepithelial smooth muscle thickness, goblet, and mast cell numbers Lower IL-25, IL-33, TSLP, caspase-3 and TUNEL	[31]
Allergic rhinitis	460 mg of herbal extract	Not animals. In AR patients	Reduced total serum IgE	[43]
	25 mg/kg	Sprague–Dawley rats	Inhibit nasal rubbing movements and sneezing	[44]
	80 mg/kg	Sprague–Dawley rats	Suppressed of AR Weaker COX-2 and VIP expressions	[45]
Atopic dermatitis	–	Nc/Nga mouse	Suppressed angiogenesis and Th2-related cytokine expression	[53]
	10 mg	Hsd:ICR (CD-1) mice	Amelioration of the tissue damage, with a noticeable attenuation of edema and leukocyte infiltration	[54]
	2 mg	Nc/Nga mouse	Decreased the iNOS and COX-2 mRNA expressions in the skin as well as significantly suppressed the increase in the level of total plasma IgE and eosinophils Down-regulated the expression of the cytokines, IL-4, 5 and 13	[50]
	–	Nc/Nga mouse	Weakened the development of AD-like skin lesions Inhibited hyperkeratosis, parakeratosis, acanthosis, mast cells and infiltration of inflammatory cells Down-regulated cytoplasmic HMGB1, RAGE, nuclear p-NF-κB, p-extracellular signal-regulated kinase (ERK) 1/2, COX2, TNFα, IL-1β, IL-2Rα, IFNγ and IL-4 and up-regulated nuclear Nrf2	[55]

*sRaw* specific airway resistance, *LAR* late-phase response, *IAR* immediate phase response, *BALF* bronchoalveolar lavage fluid, *TSLP* thymic stromal lymphopoietin, *VIP* vasoactive intestinal polypeptide, *COX* cyclooxygenase, *Nrf2* nuclear factor erythroid 2-related factor 2, *HMGB* high mobility group box, *RAGE* receptor for advanced glycation end product, *NF*-*κB* nucleartranscription factor kappa B

#### In vivo and in vitro studies

Most in vivo and in vitro studies also have investigated the effect of quercetin on leukocyte and smooth muscle contraction. In 2006, Nanua et al. have hypothesized that quercetin blocks airway epithelial cells chemokine expression via phosphatidylinositol (PI) 3 kinase-dependent mechanism. They showed that quercetin (3,3,4,5,7 pentahydroxy flavone) blocks the airway epithelial cell IL-8 and monocyte chemoattractant protein (MCP)-1 expression by attenuating the signaling through a PI-3 kinase/Akt/NF-κB pathway and also inhibits chemokine expression via transcriptional and posttranscriptional ways. Quercetin inhibits allergen sensitization, induced MCP-1 expression, and airways hyperresponsiveness, in vivo [32]. In 2011, the acute effect of quercetin on experimental allergic asthma after single-dose oral administration in vivo and in vitro was investigated by Joskova et al. They showed that quercetin (20 mg/kg) caused significant bronchodilation, both in vivo and in vitro. They concluded that quercetin could prove its ability to reduce the hyperreactivity of airways in laboratory conditions as one of the main features of allergic asthma [6]. In 2012, the effect of quercetin on mast cell activation in vitro and in vivo has been investigated by Cruz et al. They demonstrated that treatment with quercetin decrease allergen-induced development of airway hyperresponsiveness, TH2 responses in the lung, lung eosinophilia, and goblet cell metaplasia after allergen exposure to sensitized host [33]. The effect of quercetin on cytokine levels have been investigated in two studies. In 2013, Sakai-Kashiwabara et al. studied the influence of quercetin on eosinophil functions. The first set of experiments was undertaken to examine whether quercetin could suppress eosinophilia and IgE hyperproduction induced by *Mesocestoides corti* infection in BALB/c mice. Quercetin exerts suppressive effects on eosinophil activation, but not eosinophil growth and IgE hyperproduction. Therefore, quercetin will be a useful supplement for the management of eosinophil-mediated diseases, such as AR and asthma [34]. In 2015, the effect of quercetin on cytokine levels and smooth muscle contraction, in vitro and its therapeutic potential effect on a murine model of asthma have been investigated by Oliveira et al. The study shows the reduction of inflammatory cytokines production, tracheal ring relaxation and also reduction of the total number of cells in BALF and eosinophil peroxidase in the lungs by treatment with quercetin. Quercetin is potentially active as anti-asthmatic drugs, they also have both immunomodulatory and bronchodilatory properties [35]. In 2013, Townsend et al. hypothesized that quercetin relaxes airway smooth muscles (ASM) via cAMP-mediated pathways and also amplifies ß-agonist relaxation. In in vitro assays, quercetin directly attenuated phospholipase C (PLC) activity, decreased inositol phosphate synthesis, and decreased intracellular calcium responses to Gq-coupled agonists (histamine or bradykinin). Finally, the nebulization of quercetin (100 μM) in an in vivo model of airway responsiveness that increases airway resistance. Quercetin represented an entirely novel therapeutic option in the treatment of asthma due to its activity as both an inhaled phosphodiesterase 4 (PDE4) and also PLCB inhibitor with acute bronchodilator properties. Quercetin may has beneficial effects on relaxing ASM during an acute exacerbation while using alone or in combination with existing therapies such as short-acting ß-agonists. Additionally, in combination with recent work details about anti-inflammatory effects, quercetin may have been potential as an asthma therapy when using daily to prevent exacerbations [36]. In 2017, the effects of several herbs have been investigated by Luo et al. The study shows that quercetin could be used to develop new bronchodilators to treat obstructive lung diseases such as asthma and chronic obstructive pulmonary disease [37] (Table 3). Different effects of quercetin such as, inhibitory effect on mast cell activation, eosinophil activation, relaxation of tracheal ring, reduction in IL-4 and IgE serum, blocking airway epithelial cell IL-8 and MCP-1 expression suggest a valuable role of quercetin for allergic asthma. It is also suggested that quercetin might be a therapeutic candidate for allergic asthma and provide new insight into the immunopharmacological role of quercetin.

**Table 3:** Summary of main effects of quercetin on allergic diseases (in vitro and in vivo studies)

Diseases	Dosage	Cell/cell line	Animal	Quercetin’s effect	References
Allergic asthma	0.1–25 µM	16HBE14o	BALB/c	Blocks airway epithelial cell IL-8 and MCP-1 expression by attenuating the signaling through a PI-3 kinase/Akt/NF-κB pathway Inhibits chemokine expression Inhibits allergen sensitization, induced monocyte chemoattractant protein (MCP)-1 expression, and airways hyperresponsiveness, in vivo	[32]
	20 mg/kg	Spinal cord-tracheal smooth muscle	Male guinea pig	Caused significant bronchodilation, both in vivo and in vitro	[6]
	30 mg/kg	Bone marrow-derived mast cells	BALB/c	Decrease allergen-induced development of airway hyperresponsiveness, TH2 responses in the lung, lung eosinophilia, and goblet cell metaplasia	[33]
	Various dose Min–max (5–20 mg/kg)	Eosonophil	BALB/c	Suppressive effects on eosinophil activation	[34]
	30 mg/kg	Spleen-tracheal smooth muscle	A/J mice	Reduction of inflammatory cytokines production, tracheal rings relaxation and also reduction of the total number of cells in BALF and eosinophil peroxidase in lungs	[35]
	100 μM	Human ASM cells	A/J mice	Attenuated PLC activity, decreased inositol phosphate synthesis, and decreased intracellular calcium responses to Gq-coupled agonists in vitro Increase of airway resistance in vivo	[36]
	–	Human ASM cells	BALB/c	Treat obstructive lung diseases such as asthma and chronic obstructive pulmonary disease	[37]
Allergic rhinitis	20 mg/kg	HNEpC	BALB/c	Inhibited nasal symptoms and increased TRX levels in nasal lavage fluids	[47]
Atopic dermatitis	10 mg/ml	Skin biopsies	Hsd:ICR (CD-1) mice	Prevented the formation of skin lesions abrogating the various biochemical processes that cause epithelial loss and skin damage	[56]

*MCP* monocyte chemoattractant protein, *PLC* phospholipase C, *TRX* thioredoxin, *BALF* bronchoalveolar lavage fluid

## Allergic rhinitis (AR)

AR is a chronic inflammatory IgE-related disorder of the respiratory tract. AR has a high prevalence that affects up to 40% of the population, which results in labor loss, impaired quality of life, and comorbid diseases [38–40]. Numerous inflammatory cells, including mast cells, CD4^+^ T cells, B cells, macrophages, and eosinophils, infiltrate the nasal lining in AR patients after exposure to an inciting allergen (most commonly airborne dust mite fecal particles, cockroach residues, animal dander, molds, and pollens). In allergic individuals, the T cells which are infiltrating the nasal mucosa are predominantly Th2 in nature and release cytokines (e.g., IL-3, IL-4, IL-5, and IL-13) that promote IgE production by plasma cells. Crosslinking of IgE bound to mast cells by allergens, in turn, triggers the release of mediators, such as histamine and leukotrienes that are responsible for arteriolar dilation, increased vascular permeability, itching, rhinorrhea, mucus secretion, and smooth muscle contraction in the lung. The mediators and cytokines released during the early phase of an immune response to an inciting allergen trigger a further cellular inflammatory response over the next 4–8 h (late-phase inflammatory response) resulting in recurrent symptoms (usually nasal congestion) that often persist [40–42]. A lot of medical treatment modalities used as a treatment of AR, such as antihistamines, steroids, montelukast (Singulair), and immunotherapy. However, these therapeutic modalities can fail on some occasions [42]. Thus, in vitro and in vivo studies have been managed to make AR models and these studies also assess the effects of different agents such as quercetin.

### Effect of quercetin on AR

#### In vitro studies

Two in vitro studies have investigated the effect of quercetin on human nasal epithelial cells (HNEpC). The quercetin influences on the production of both substances including periostin, a 90-kDa extracellular matrix protein that is attracting attention as a novel biomarker of airway inflammatory diseases such as AR and asthma, and periostin-induced eosinophil chemoattractants from HNEpC have been examined by Irie et al. Treatment of HNEpC with quercetin at a concentration of 4.0 μM suppressed the ability of cells to produce CC-chemokine ligand 5 (CCL5) and eotaxin. These results showed that quercetin can suppress the production of both periostin and periostin-induced eosinophil chemoattractants from HNEpC and results in the improvement of the clinical condition of AR [38]. In 2018, the influence of quercetin on nitric oxide (NO) production from HNEpC after IL-4 stimulation has been investigated by Ebihara et al. The results significantly remarked that quercetin modified the clinical condition of RA through the suppression of NO production from nasal epithelial cells after IL-4 stimulation. The minimum concentration of quercetin that caused significant suppression was 1.0 nM [41] (Table 1). It can be suggested that quercetin has a valuable effect on AR by influencing on HNEpC. More in vitro studies is required to clarify the exact role of quercetin on immune cells in AR patients.

#### In vivo studies

Increased levels of IgE are a hallmark of allergic diseases. A proper treatment for allergic diseases should be able to reduce serum IgE levels. In 2001, the effects of Biminne, a kind of Chinese herbal formulation in patients struggling with moderate to severe perennial AR have been determined by Hu et al. Quercetin and baicalein are two major flavonoids in Biminne. A pilot dose–response study showed both half (230 mg of herbal extract) and full (460 mg of herbal extract) strengths were effective. Total serum IgE was reduced after the herbal treatment. The results suggest that the Biminne formulation is effective in the treatment of perennial AR. The mechanism of action is unknown [43]. Reduction in the symptoms of AR has been demonstrated in the study of Kashiwabara et al. They examined the influence of quercetin on the development of AR by using Sprague–Dawley rats. The results demonstrated that oral administration of quercetin for 5 and 7 days could inhibit nasal rubbing movements and sneezing. The minimum dose that caused significant inhibition was 25 mg/kg. The results strongly suggested that quercetin will be well-qualified as a supplement for the management and treatment of allergic diseases, especially AR [44]. In 2017, Sagit et al. have been noticed that quercetin had a therapeutic effect on an experimental rat model of AR. They observed that AR was suppressed in the quercetin (dose: 80 mg/kg) group comparing to the control group. In immune histochemical evaluation, it was detected that COX-2 and vasoactive intestinal polypeptide (VIP) expressions were weaker in the quercetin group than the control group. According to these findings, they concluded that quercetin was effective in AR-induced by OVA in rats both histopathologically and serologically [45] (Table 2).

#### In vitro and in vivo studies

Thioredoxin (TRX) is a protein that regulates reactive oxidative metabolism and scavenges reacting oxygen species, which is implicated in the mechanism of asthma. Some studies have shown that TRX suppresses allergic inflammation [46]. In 2018, the effects of quercetin on AR symptoms and the role of the TRX production of nasal epithelial cells in vitro and in vivo have been investigated by Edo et al. The results showed that the oral administration of 20 mg/kg of quercetin significantly inhibited nasal symptoms and the same dose of quercetin significantly increased TRX levels in nasal lavage fluids. Quercetin’s ability to increase TRX production may be useful, at least in part, for its clinical efficacy toward AR [47] (Table 3). Similar to asthma allergic, quercetin seems to be a good therapeutic candidate for AR. However, the study of quercetin on AR is lower than asthma allergic. Similar to studies on asthma allergic, the dose of 25 mg/kg quercetin seems to be enough for inhibiting the symptoms in AR.

## Atopic dermatitis (AD)

AD is chronic, inflammatory skin disease, with a prevalence of up to 7% in adults and up to 25% among children. Different mechanisms including environmental, psychological, immunological, pharmacological, and genetic factors play role in AD pathogenesis. In fact, 31 risk loci associated with AD have been identified. Characteristically, symptoms start within the first 5 years of life, and in adults, the disease has generally been present for decades [48, 49].

AD is considered a primarily T cell-driven disease as proved by the clinical efficacy of broad T cell–targeting therapeutics, such as cyclosporine, efalizumab, and alefacept. Although efalizumab and alefacept are no longer available because of safety concerns, cyclosporine, oral glucocorticoid steroids, and phototherapy (narrow-band UVB) are often used to treat moderate-to-severe disease. However, cyclosporine and, even more so, glucocorticoid steroids are not suitable for long-term use because of multiple side effects. Phototherapy is too time consuming and not feasible for most patients. Therefore, AD presents a large unmet need for both effective and safe therapeutics [49, 50]. Based on side effects of therapeutic agents which are used for AD, several studies have investigated the effects of quercetin in AD.

### Effect of quercetin on AD

#### In vitro studies

Two studies have examined the effect of quercetin on heme oxygenase (HO) and oxidative stress. In 2009, the role of HO-1 in the anti-allergic action of quercetin against the degranulation of rat basophilic leukemia (RBL-2H3) cells, rat peritoneal mast cells, and mouse bone marrow-derived mast cells has been investigated by Matsushima et al. HO activity was upregulated after short exposure to quercetin, followed by the induction of HO-1 expression after long exposure to quercetin. The results strongly suggest that quercetin exerted anti-allergic actions via activation of nuclear factor erythroid 2-related factor 2 (Nrf2(-HO-1 pathways [51]. The improvement of quercetin protective effect against oxidative stress skin damage by incorporation in nanovesicles has been evaluated by Manca et al. Quercetin was taking part in glycerosomes, new phospholipid-glycerol vesicles, and their protective effect against oxidative stress skin damages has been assessed. Quercetin incorporated into liposomal and glycerosomal nanoformulations showed a strong ability to scavenge free radicals and protect human keratinocytes in vitro against hydrogen peroxide damage. Moreover, quercetin-loaded vesicles were avidly taken up by keratinocytes in vitro. Overall, results indicate 40 and 50% glycerosomes as promising nanosystems for the improvement of cutaneous quercetin delivery and keratinocyte protection against oxidative stress damage [52] (Table 1).

#### In vivo studies

The effect of quercetin on Th2-related cytokines which are crucial in allergic diseases has been studied by Jung et al. in 2010. They reported that tannic acid (TA) and quercetin suppressed angiogenesis and Th2-related cytokine expression including TSLP and thymus and activation-regulated chemokine (TARC), in an AD-like Nc/Nga mouse model. Furthermore, they have a therapeutic effect on AD. TA and quercetin might be effective and improved therapeutic uses that should be investigated further for the treatment of AD [53]. In 2013, Caddeo et al. developed quercetin-loaded phospholipid vesicles, named liposomes and Penetration Enhancer-containing Vesicles (PEVs), and investigated the efficacy on tetradecanoylphorbol 13-acetate (TPA)-induced skin inflammation. The administration of vesicular quercetin on TPA-inflamed skin resulted in an amelioration of the tissue damage, with a noticeable attenuation of edema and leukocyte infiltration, especially using 5% PEG-PEVs, confirmed by confocal microscopy. So, the proposed approach based on quercetin vesicular formulations may be valuable in the treatment of inflammatory skin disorders [54]. In 2014, Park et al. isolated quercetin-3-O-(2″-gallate)-a-l-rhamnopyranoside (QGR) from the leaves of a native plant of Korea named Acer ginnala Maxim, and they evaluated the anti-inflammatory and anti-allergic effect of QGR in a murine model of AD. Topical QGR significantly decreased the iNOS and COX-2 mRNA expressions in the skin as well as significantly suppressed the increase in the level of total plasma IgE and eosinophils. In addition, topical application of QGR down-regulated the expression of the cytokines, such as IL-4, 5 and 13, which were induced by *Dermatophagoides farina* ointment stimulation. So, the results demonstrate that QGR might be beneficial in the treatment of AD [50]. In 2015, the effects of quercetin on skin lesion, high mobility group box (HMGB) 1 cascade signaling and inflammation in the AD mouse model have been investigated by Karuppagoundera et al. AD-like lesion was induced by the application of house dust mite extract to the dorsal skin of NC/Nga transgenic mice. Quercetin treatment weakened the development of AD-like skin lesions. Histological analysis showed that quercetin inhibited hyperkeratosis, parakeratosis, acanthosis, mast cells and infiltration of inflammatory cells. Furthermore, quercetin treatment down-regulated cytoplasmic HMGB1, receptor for advanced glycation end product (RAGE), nuclear p-NF-κB, p-extracellular signal-regulated kinase (ERK) 1/2, COX2, TNFα, IL-1β, IL-2Rα, IFN-γ and IL-4 and up-regulated nuclear Nrf2. Their data indicated that the HMGB1/RAGE/NF-κB signaling might play an important role in skin inflammation, and quercetin treatment could be a promising agent for AD by modulating the HMGB1/RAGE/NF-κB signaling and induction of Nrf2 protein [55] (Table 2).

#### In vitro and in vivo studies

In 2014, Castangia et al. developed biocompatible quercetin and curcumin nanovesicles as a novel approach to prevent and restore skin tissue defects on chronic cutaneous pathologies. Their results showed that nano entrapped polyphenols prevented skin lesions formation abrogating the various biochemical processes that cause epithelial loss and skin damage [56]. The effects of quercetin on AD seem to be similar to other allergic diseases. It suggested that quercetin affects different immune and non-immune cells in the same pathway (Table 3).

## Conclusion

In the current article, we reviewed the effect of quercetin on allergic asthma, AR, and AD. Allergic diseases are a big concern and have high health care costs. In addition, the use of current therapies such as ß2-agonists and corticosteroids has been limited for long term use due to their numerous side effects. Quercetin, which has a long story of usage in human history, has been demonstrated sufficient efficacy and has no significant side effects. It has the potential to reduce the most significant pathologies of asthma such as eosinophil and neutrophil recruitment, the activation of bronchial epithelial cells, collagen and mucus production and airway hyperactivity. It also can suppress the production of both periostin and periostin-induced eosinophil chemoattractants and resulting in the improvement of the clinical condition of AR. In fact, it will be a good candidate as a supplement for the management and treatment of allergic diseases, especially rhinitis. Since medicinal plants have a low price, natural origin, and fewer side effects, quercetin seems to be a good therapeutic nominee for allergic diseases in clinical trials.

## Data Availability

Not applicable.

## Abbreviations

BALF: Bronchoalveolar lavage fluid
COX: Cyclooxygenase
EGFR: Epidermal growth factor receptor
ERK: Extracellular-regulated kinase
HMGB: High mobility group box
HNE: Human neutrophil elastase
HNEpC: Human nasal epithelial cells
HO: Heme oxygenase
IAR: Immediate phase response
LAR: Late-phase response
MCP: Monocyte chemoattractant protein
MUC5AC: Mucin 5 AC
NF-κB: Nucleartranscription factor kappa B
NO: Nitric oxide
Nrf2: Nuclear factor erythroid 2-related factor 2
PKC: Protein kinase C
PLC: Phospholipase C
RA: Allergic rhinitis
RAGE: Receptor for advanced glycation end product
RANTES: Regulated on activation, normal t cell expressed and secreted
sRaw: Specific airway resistance
TRX: Thioredoxin
TSLP: Thymic stromal lymphopoietin
VIP: Vasoactive intestinal polypeptide
